# Remembering the Personal Past: Beyond the Boundaries of Imagination

**DOI:** 10.3389/fpsyg.2020.585352

**Published:** 2020-09-30

**Authors:** Christopher Jude McCarroll

**Affiliations:** Centre for Philosophy of Memory, Université Grenoble Alpes, Grenoble, France

**Keywords:** episodic memory, episodic imagination, causal theory of memory, simulation theory of memory, forgetting, childhood amnesia

## Abstract

What is the relation between episodic memory and episodic (or experiential) imagination? According to the causal theory of memory, memory differs from imagination because remembering entails the existence of a continuous causal connection between one’s original experience of an event and one’s subsequent memory, a connection that is maintained by a memory trace. The simulation theory rejects this conception of memory, arguing against the necessity of a memory trace for successful remembering. I show that the simulation theory faces two serious problems, which are better explained by appealing to a causal connection maintained by a memory trace. Remembering the personal past is not the same as imagining.

how do we remember, and what is it we find in the end?

–W. G. Sebald, *Austerlitz*

## Introduction

Writing in his autobiography, a book about his “secret life,” the Spanish artist Salvador Dalí makes an assertion about personal memory^1^. He declares:

I presume that my readers do not at all remember, or remember only very vaguely, that highly important period of their existence which anteceded their birth and which transpired in their mother’s womb. But I-yes, I remember this period, as though it were only yesterday… (these) memories, so rare and liquid, which I have preserved of that intra-uterine life … will undoubtedly be the first of this kind in the world since the beginning of literary history to see the light of day and to be described systematically (Dalí, 1942/1993: Ch. 2).

What are we to make of Dalí’s claim to “intra-uterine” memories? Are these memories genuine? Or are they the whimsical products of a famously creative mind? What Dalí is describing here seem to be rich and powerful fantasies of his past rather than genuine memories. After all, we typically forget and cannot recall moments from our very early life. Indeed, can we even be said to experience these intra-uterine moments in any real robust sense (Lagercrantz, 2014)?

But, then, what if memory just *is* imagination? What if to episodically remember a past event is merely to imagine that episode in one’s personal past? Would Dalí’s memories, “so rare and liquid,” count as genuine?

This paper explores recent claims that episodic memory is merely one form of episodic (or experiential) imagination (Michaelian, 2016a; Hopkins, 2018)^2^. Roughly, on such a view, episodic imagination is our ability to mentally entertain possible episodes. Episodic memory, then, is simply one form of this imaginative capacity in which we mentally revisit or re-enact episodes in our personal pasts. Given that there are many different kinds of imagining, a more precise definition of the type of imagining that remembering is supposed to be is warranted. Addressing the debate about the relation between memory and imagination, Langland-Hassan (Forthcoming) concludes that the type of imagining involved is best described as *constructive imagining*, which refers to “the capacity to form novel representations” (Van Leeuwen, 2013, p. 204). Saying that memory just is imagination is to say that memory is constructive imagination (Langland-Hassan, Forthcoming).

Both (episodic) imagination and memory tend to have a rich phenomenology, replete with visual and spatial mental imagery and emotion, for example. We *see* and *feel* these events unfold before our minds. This points to a certain similarity between the two processes. Yet, the claim that memory is imagination is a significant one, and it has important theoretical implications for what we think memory is and how it works. Indeed, the claim that episodic memory is merely a form of episodic imagination, at least on some of its characterizations, seems to lead to some improbable attributions of memory–attributions such as Dalí’s claim to remember the period before his birth. This creates a problem because it seems to undermine the idea that episodic memory depends on one’s experience of a past event.

According to the influential causal theory of memory (Martin and Deutscher, 1966), memory differs from imagination in that genuine remembering presupposes an appropriate causal connection between the subject’s current representation of an event and her earlier experience of it. This appropriate causal connection is one that is maintained by a memory trace. There is no widely accepted definition of a memory trace in the literature, but the general idea is that memory traces bridge the gap between a past event and one’s current memory experience of that past event (De Brigard, 2014b; Robins, 2017; Werning, 2020). Information about an event is encoded in a memory trace, which preserves this information over time and makes it available for subsequent retrieval. There are many different conceptions of memory traces, and different views as to the role they play in remembering (Robins, 2017). The causal theory of memory operates with a physical notion of a memory trace, which stores information over time and is causally operative in producing the present memory representation. Revising the classical causal theory’s notion of traces somewhat, they can be thought of as “structural modifications at synapses…that affect the ease with which neurons in a neural network can activate each other” (Bernecker, 2010, p. 132), and which store information about the event in a distributed manner (Sutton, 1998; cf. Robins, 2016). Even though imaginings (and false memories) may also involve representations that are constructed from stored information, a memory trace links the memory representation back to the single episode that is recalled. The notion of a memory trace is key to the causal theory and distinguishes genuine memory from mere imagining (cf. Langland-Hassan, Forthcoming).

The causal theory is somewhat in tension with empirical evidence that remembering is a constructive and reconstructive process, however^3^. And, although the causal theory has been modified to accommodate this evidence (Michaelian, 2011a), it has recently come under fire from a new theory of remembering–the simulation theory (Michaelian, 2016a)–which interprets this evidence in a different light, arguing that it forces us to abandon the causal condition on memory. On the simulation theory, episodic remembering is a form of imagining.

I suggest that this reinterpretation of the evidence is a step too far. I show that the simulation theory faces serious challenges. One of the main virtues of the simulation theory is that it seeks to provide an empirically informed account of memory. Yet, I show that the simulation theory, in its current articulation, fails to account for two important and ubiquitous features of human remembering. I show that rejecting the idea that memory involves a causal connection to the past event has counterintuitive consequences and leads to some implausible attributions of memory. Remembering is not the same as imagining.

## Remembering the Personal Past: Causal Connections, or Imaginative Simulations?

According to the causal theory (Martin and Deutscher, 1966), remembering is a distinct kind of state or process to imagination. Genuine memories are appropriately causally connected to the events that they are about^4^. On this view, genuine remembering requires a continuous causal connection in the form of a memory trace^5^. The memory trace has to be continuously stored and play a causal role in generating the memory representation at the time of retrieval. This is the key insight of the causal theory of memory. For genuine remembering, “it is necessary that a relevant event of information acquisition has left some “trace,” a trace which has been preserved and is causally relevant for the occurrence of the mental state or event which should count as a memory” (Debus, 2017, p. 68)^6^. This causal connection in the form of a memory trace ensures that remembering is a different kind of state or process to imagination and merely relearning previously attained information.

On the causal theory, memory can be understood as a *diachronic* capacity, because for “remembering to occur is for there to be a particular relationship between representations located at two different points in time” (Michaelian and Robins, 2018, p. 14). In other words, content gained at a time in the past is stored and then transmitted to the memory representation in the present. The idea that all the content is stored in the form of a trace is challenged by the evidence about the inherently constructive nature of episodic memory. Individuals often incorporate information that was not available at the time of the original event into their memory representations (Suddendorf et al., 2009), and, in some cases, people can come to generate entirely false memories, seeming to recall events that did not in fact take place (Loftus, 1993). To remember is not to retrieve a representation of a past event in the form of a memory trace, but rather to generate a representation that may incorporate content that was unavailable at the time of the original event and hence was not stored in a trace. Even though the classical causal theory is in tension with the reconstructive nature of remembering, the causal theory can be updated and modified to reflect memory’s creativity (Michaelian, 2011a). On the causal theory of constructive memory, content that was not encoded in the original trace can become part of the trace at a later point in the memory process, or part of the content of the memory representation at retrieval.

Perhaps, though, the causal theory of constructive memory does not go far enough. The simulation theory rejects the necessity of an appropriate causal connection for successful remembering. On the simulation theory, episodic memory is simply a form of imagination (Michaelian, 2016a, 2018). On this view, episodic remembering is imagining or simulating an event in one’s personal past. Successful remembering occurs when the simulation of the past event is produced by a reliably (properly) functioning episodic construction system, a neurocognitive system which also constructs simulations of other scenarios such as future and counterfactual episodes (De Brigard, 2014a).

There is evidence for a single cognitive mechanism for a range of imaginative processes from the evidence that memory is a form of “mental time travel” (MTT) (Tulving, 1985). According to the MTT framework, remembering the past (episodic memory) and imagining the future (episodic future thinking) are different temporal dimensions of the same capacity to mentally travel in time (Michaelian et al., 2016). The function of remembering may be to generate representations of possible events in the personal future, or even simulate hypothetical scenarios, rather than faithfully replaying the past (Schacter and Addis, 2007; De Brigard, 2014a). A key motivation for developing the simulation theory is this empirical support for the MTT framework. This empirical evidence includes imaging, behavioral, clinical, and developmental data, all of which show important connections between remembering the past and other imaginative states or processes. On the simulation theory, episodic memory is part of a more general capacity to engage in episodic imagination (Michaelian, 2016a; Addis, 2018).

For the simulation theorist, the constructive processes in remembering, combined with the evidence on mental time travel regarding the connection between memory and imagination, mean that we have to give up the causal theory of (constructive) memory. For the simulation theorist, the causal theory of constructive memory, where an appropriate degree of change in content between the past and present representations can occur, is much too vague: it is unclear how much similarity in content is required, which dimensions of similarity are relevant (Michaelian, 2016a, p. 91). Further, the empirical evidence on construction and mental time travel suggests that there is often simply too much deviation in content between a past experience and a present memory for the causal theory to account for. For the simulation theorist, “the only factor that distinguishes remembering an episode from merely imagining it is that the relevant representation is produced by a properly functioning episodic construction system … which aims to simulate an episode from the personal past” (Michaelian, 2016a, p. 97)^7^.

It is important to note that the simulation theory does not deny that information may be retained in the form of a memory trace. Even on the simulation theory, many cases of remembering will involve the sort of continuous causal connection described by the causal theory. The key idea is, rather, that this causal connection is not necessary for successful remembering. Successful remembering may draw on other sources of information and, at least in some cases, “one can remember without drawing on information originating in one’s experience of the remembered episode” (Michaelian, 2016a, p. 118). This is one of the key claims of the simulation theory, and it is a bold one. A further important, and perhaps bolder, claim advocated by the simulation theory, is that because the personal past may include non-experienced events, perhaps because one was too young to properly count as experiencing them, then “one can in principle remember an entire episode that one did not experience” (Michaelian, 2016a, p. 118). As long as one is representing an event in one’s personal past, and this representation is constructed by a reliably functioning episodic construction system, then one is remembering^8^. In this sense, and in contrast to the causal theory, the simulation theory offers a *synchronic* understanding of the content of episodic memory: content is not something that is gained in the past, stored, and then transmitted, but rather all the content that is required for the construction of a memory representation can be acquired in the present at a synchronic moment in time (Michaelian and Robins, 2018)^9^.

These two claims–memory need not draw on any information originating in the original event, and one can remember non-experienced events–lie at the heart of the simulation theory. It is these two claims that most fully distinguish the simulation theory from the causal theory of constructive memory. Yet, as Michaelian himself acknowledges, both claims, especially the second, will be “hard to swallow” for many people (Michaelian, 2016a, p. 118). In the rest of the paper I look at problems for the simulation theory that arise from its commitment to these claims.

## Remembering Forgetting

Forgetting is the counterpart to remembering. Forgetting is a ubiquitous feature of human memory. Even in the absence of cognitive decline, we routinely forget many events in our lives, especially those from our early childhoods (see section “Recalling Non-experienced Events”). Forgetting intuitively involves the loss of “mental items” (Caravà, 2020). On the casual theory, forgetting occurs when stored information is no longer available, or when we lose access to some stored information, where this stored information is thought of as a memory trace. As Michaelian himself acknowledges in an earlier paper about the two components of forgetting: “We can distinguish between forgetting in the sense of the permanent elimination of a memory trace (the unavailability of a record) and forgetting in the sense of the (possibly temporary) inaccessibility of a trace” (Michaelian, 2011b, p. 403)^10^. So forgetting seems to involve the absence of stored content, not being able to retrieve, for whatever reason, some information in the form of a memory trace.

Forgetting can also be about the details. We routinely remember particular events, even though some of the context or other details of the event are not recalled. Indeed, this loss of contextual information may explain the semanticization of episodic memory over time. Whereas episodic memory represents events that were personally experienced, semantic memory is our memory for facts or conceptual knowledge about the world, including events that were not personally experienced (Tulving, 1985). For Mark Rowlands, the difference between episodic memory and semantic memory is one of degree. Episodic memories, which are rich in experiential content (e.g., emotional, visual, and kinesthetic information), gradually lose this content over time. If, Rowlands tells us, “this process of experiential denuding continues long enough, then what is left at its end is clear. I am left with a semantic memory” (Rowlands, 2016, p. 43). So, even though we can gain semantic memories by learning facts about the world, as time passes, specific episodic memory content can be lost and memory can become semanticized. Even if the difference between episodic and semantic memory does not lie in a diminution of content, but perhaps the manner in which content is experienced at retrieval (Klein, 2015), the key point I am making still holds: episodic memories frequently lose sensory and contextual details over time, such that the content of memory is less detailed for temporally remote events (D’Argembeau and Van der Linden, 2004).

There are some people who seem to have prodigious memories. Individuals who can remember an extraordinary amount of detail from their personal pasts, recalling the days of the week that particular dates fell on and providing detailed information about what happened on those days. Such individuals can remember “details of what happened…from every day of their life since mid-childhood” (Patihis et al., 2013, p. 20947). Such individuals have hyperthymesia, or Highly Superior Autobiographical Memory (HSAM). But this is a “rare ability,” and less than 100 people with HSAM have been discovered (McGaugh, 2017). It is extraordinary rather than ordinary, and it is not the norm for human memory and forgetting. Further, it is important to note that even individuals with HSAM forget some aspects of their personal pasts. Discussing the first reported case of an individual with HSAM, McGaugh and colleagues inform us that “her autobiographical memory, while incredible, is also selective and even ordinary in some respects” (Parker et al., 2006, p. 42).

Forgetting is a hence pervasive feature of the normal memory process. We remember fewer events and in less detail than what we experienced. Yet the simulation theory seems to downplay the importance of forgetting to the normal memory process. Both these forms of forgetting—the forgetting of whole episodes and the forgetting of contextual details—seem to imply that remembering involves a trace. As we saw, the simulation theorist does not deny that traces are sometimes involved in memory, and forgetting may even be understood as an important part of a reliably functioning episodic memory system (Michaelian, 2011b). But if remembering is simply imagining an event in one’s personal past, then as long as one has access to information about that past event (e.g., from photographs or third-person testimony) one could, in principle, *always* remember one’s past. At present, the only explanation of forgetting that the simulation theory has offered is that it occurs “when the subject fails to produce a representation that he should have produced” (Michaelian, 2016b, p. 12). But if the subject potentially always has access to the information necessary for the construction of a simulation of a past event, then barring a total failure of a properly functioning episodic construction system, the subject could *always* succeed in producing a representation of the past event. There is *potentially* no forgetting if remembering is merely (reliably) imagining. But this is a highly counterintuitive consequence of the simulation theory^11^.

Even if we do not want to consider forgetting as a memory error (Michaelian, 2018), it is still something that a theory of episodic memory should account for Frise (2018). By postulating a causal connection to one’s past, a memory trace, which can, through age, injury, or neglect, be disrupted or destroyed, we have a relatively straightforward way of explaining the counterpoint to memory that is forgetting.

One of the simulationist motivations to move away from the causal theory was because of its inherent vagueness about the similarity required between the content of the past experience and the content of the present memory. Let us call this “trace vagueness.” But if the simulation theorist embraces the notion of traces to explain forgetting, then the problem of vagueness enters at the other extreme, at the point of retrieval. One of the most striking claims of the simulation theory is that memory need not draw on any information originating in the original event. But if we want to acknowledge that forgetting routinely occurs, how many cases of genuine remembering will involve no content originating in the original event? The simulationist answer to this is not clear. Let us call this “retrieval vagueness.” If the simulationist appeals to traces to explain routine and pervasive forgetting, then there is a vagueness inherent in the theory about when remembering will actually draw on no information stored in a trace.

In fact, the simulation theory cannot simply resort to an explanation of forgetting that relies on traces. The reason lies in the simulation theory’s emphasis on the synchronic nature of content generation in episodic memory. The causal theory sees memory as a diachronic capacity, where the relation between two representations at different temporal points is important. The simulation theory views memory as a s*ynchronic* process, in which the context of retrieval is emphasized as the point of content generation. The notion of forgetting seems to presuppose that memory is a diachronic capacity. Therefore, the simulationist cannot explain forgetting by appealing to traces, because even if one may have forgotten some past event through the decay or absence of a trace, according to the simulationist one can *still* construct a genuine memory representation in the present based on other sources of information (e.g., testimony). On the simulation theory, cases of relearning information in the present can, once the representation has been internalized, subsequently count as genuine cases of remembering (see section “Recalling Non-experienced Events”). Any appeal to traces to explain forgetting is to forget the consequences of the simulationist’s synchronic understanding of the generation of episodic memory content^12^.

This is the case even if the simulationist endorses an enactivist-inspired understanding of memory traces and their role in forgetting. Enactivist accounts of the mind (broadly) reject the notion of mental representations for many forms of cognition (e.g., Hutto and Myin, 2017). The notion of stored content that is gradually forgotten over time is therefore anathema to enactivists (Hutto and Peeters, 2018). Caravà (2020) proposes a sophisticated, alternative account of forgetting by appealing to the notion of a memory trace that is compatible with the central tenets of enactivism. On this understanding, a memory trace is not a stored representation that is reactivated at recall, but is a disposition to enact or simulate some past experience of an event. These dispositions are properties that supervene on physical properties of the body and brain, and “they amount to the disposition of the brain to re-activate some pattern of activity” (Caravà, 2020, p. 8; see also De Brigard, 2014a,b). Forgetting is conceived as the temporary or permanent inaccessibility of (dispositional) memory traces.

Noting a theoretical link between enactive and simulationist approaches to episodic memory, in that they both emphasize the active, imaginative, and constructive character of remembering, Caravà suggests that her enactive solution could also help provide a simulationist account of the nature of forgetting:^13^

Just like in the enactive approach forgetting occurs by virtue of an active process in which some simulative paths become more relevant than others, it might be argued that, for the simulationists, forgetting occurs because of the competition between information originating from different experiences in the recombinatory phase of simulation (Caravà, 2020, p. 12).

This solution to the problem of forgetting does not work for the simulationist account, however. Recall that there is a particular issue at stake in the problem of forgetting for the simulation theory. On the simulation theory, a particular instance of remembering need not draw on *any* information originating in the past event: the episodic simulation can be constructed *exclusively* from information in the present. The issue of forgetting that the simulationist faces is precisely when there is *no competition* between various sources of information, when there is no memory trace (dispositional or contentful) involved in the construction of the memory. If a memory can be constructed exclusively from a source of information in the present, when we have access to all the necessary information and are attending to it, then there is no way for the mechanisms of forgetting to occur.

The simulationist owes us an explanation of forgetting that *does not* appeal to traces. If remembering is imagining, forgetting would seem to require a *global* failure of the (normally reliable) episodic construction system to generate an imaginative simulation of a past event, even though it has all the information it requires for such a construction. At present, the simulationist account of remembering has not offered a solution to the problem of forgetting.

## Recalling Non-Experienced Events

On the simulation theory one can remember non-experienced events. But this claim leads to some puzzling attributions of memory. If remembering is merely imagining an event in one’s personal past, then provided that one has access to information from some source to simulate the past event (e.g., from testimony), it seems that one could remember events during which one was unconscious, such as periods of dreamless sleep or undergoing an operation that involved general anesthesia. Can we remember such events or are we merely imagining them? I think such cases are problematic for the simulation theory, but I am going to leave the discussion of them here and concentrate on a type of case that seems closer to the kind of non-experienced event the simulation theory describes^14^.

In the eyewitness memory literature, researchers typically try to implant false memories in subjects, asking them to imagine events, events such as being lost in a mall, that are supposedly part of their childhoods but in fact never happened (Loftus, 1993). Yet, consider a scenario in which this procedure was used when, unbeknown to the researchers, the subject was actually lost in the mall as a child but was too young to count as having experienced the episode (on a broad understanding). According to the simulation theory, in such a case, “the subject does not even misremember–he simply remembers the episode. That he did not actually experience it makes no difference” (Michaelian, 2016a, p. 119).

In this case, even though the subject was too young to count as experiencing the event (being lost in the mall), he can still remember it because he has access to testimonial information about how the event unfolded and can generate a representation (simulation) of the event. But can we remember events from even earlier moments in our lives? To return to Dalí’s claim about memory, on the simulation theory would intra-uterine memories count as genuine? Is it possible to remember the moment of one’s birth?

It is well established that most people do not easily report memories from these early periods of their life. Consider the phenomenon of “infantile amnesia,” where there is ample empirical evidence to show that adults tend to fail to recall extremely early childhood events, roughly before 2–4 years of age. There are many different explanations of this phenomenon^15^. Psychological explanations appeal to an underdeveloped sense of self, theory of mind, or language. But given that an equivalent “infantile amnesia” is also observed in rodents, the phenomenon “attests to the fact that the developing neonatal brain is anatomically and functionally different from the adult brain, therefore processing and storing information should be accomplished in a manner different from that of the adult brain” (Sara, 2017, p. 2). People may be able to imagine events in their lives at such an early age, but they typically cannot recall them. Yet if remembering is merely imagining, why is infantile amnesia such a robust phenomenon?

What about on the simulation theory: could these representations of being born count as genuine memories? Can the simulation theory explain the phenomenon of childhood amnesia? When Dalí and others claim to remember such early events, they certainly appear to be using their imagination; these states are also about events in their personal pasts; and the people who report remembering these events do not seem, on the face of it, to be suffering from amnesia or memory-related problems, so they seem to have reliably functioning episodic construction systems. In this sense, these states seem to satisfy the requirements the simulation theory places on genuine remembering. But these seem to be implausible attributions of memory. There are a number of ways that the simulation theorist could reply, but, as I show, ultimately the commitment of the simulation theory to a *synchronic* understanding of episodic memory means that it lacks the resources to account for childhood amnesia.

One option would be to retreat to the claim that memory often involves a trace, and these puzzling cases are examples of states that do not maintain this link to the past and hence are not genuine memories. But this response is simply to revert to a version of the causal theory. Moreover, it seems unclear how much weight we should then give to the claim that one can, in principle, remember an event that one did not experience. Which representations of non-experienced events count as memories? How many of these imagined non-experienced events can we call memory? All of them, or only some of them? Part of the simulation theorist’s motivation to move away from the causal theory was the vagueness involved in trying to specify how similar a past and present representation must be for the latter to count as memory. But here we have vagueness enter at a different point. The simulation theory is again beset by retrieval vagueness: it is unclear how often genuine memories will be constructed exclusively in the present, at the moment of retrieval, without drawing on stored information.

If the simulation theorist wants to deny that these impossible memories are genuine, then perhaps the most convincing argument would be to suggest that these individuals do not, or more precisely did not, have a reliably functioning episodic construction system. That is, the simulation theorist could tell us that there is a developmental aspect to the episodic construction system, and that because the episodic construction system is not fully developed, then events from such a young age cannot be remembered. This actually fits with the scientific evidence: as we saw above, research “has long established that as adults we cannot accurately retrieve memories from our infancy and early childhood. To put it simply, the brains of babies are not yet physiologically capable of forming and storing long-term memories” (Shaw, 2016, p. 3). But if the simulation theorist acknowledges this developmental aspect, then this places the emphasis on forming (encoding) and *storing* memories, which is a key component of the causal theory. This is in tension with the key claims of the simulation theory. The simulation theory is *synchronic* in that it emphasizes the role that retrieval plays in the memory process, and if it acknowledges the importance of encoding and storage in this way, then it reverts to a diachronic understanding of how the content of episodic memory is constructed. If stored content (or even dispositional traces) does not matter for genuine memories of non-experienced events, then at the very least there is a tension between the simulation theory’s key claims and the appeal to stored content to explain these puzzling examples.

Perhaps another response is to say that these cases do not satisfy an “internality” condition on memory. In order to distinguish between remembering and relearning, whereby a person forms a memory representation of an event based solely on external sources of information, Michaelian (2016b) introduces the condition of “internality,” whereby in remembering the subject contributes some information to the representation. In contrast, (veridical) “relearning occurs in cases in which the subject seems to remember, and to remember accurately, but in which he himself contributes no content to the retrieved memory representation” (Michaelian, 2016b, p. 10). Perhaps the puzzling cases of remembering being born can be explained away because they do not satisfy the internality condition? I do not think this is quite right, however. Even if internality is an appropriate condition to distinguish remembering from relearning in general, it fails in this case. First, failure to satisfy the internality condition is very specific, and relates only to the precise time of relearning. Second, the internality condition does not mean that the subject must contribute information stored in a trace, but only that the subject contribute information. The people who recall these “memories” seem to imaginatively fill in missing details and so they are satisfying the internality condition. Finally, even if a (seeming) memory representation is based fully on external information, and hence is relearned, this information can then be internalized such that the subject can be said to remember. Even if these states were initially based wholly on external information (relearned), on the simulation theory the representations will count as memories because the information has been subsequently internalized.

In a sense this might be the most appropriate response for the simulation theorist: to simply accept that these “memories,” no matter how puzzling, are genuine. This response would seem to be the most consistent with the theory. But, as we saw, it also does not seem to fit the science and, if anything, the simulation theory seeks to be empirically informed. In fact, in a similar way to the problem of forgetting outlined above (see section “Remembering Forgetting”), given the pervasiveness of childhood amnesia, the simulation theory owes us an explanation of why it occurs. The simulation theory does not seem to have the resources to explain childhood amnesia. If the simulation theory appeals to traces to explain why individuals typically cannot recall events from a very early age, this is only to postpone the explanation of childhood amnesia. If remembering is a synchronic endeavor, then there is *always* the possibility of remembering any event in one’s life. Traces can help provide an explanation of childhood amnesia, but the simulation theory goes beyond traces. On the simulation theory genuine remembering is *always* possible even without a memory trace. Again, if remembering is merely imagining, why is infantile amnesia such a robust phenomenon? Why can more people simply not remember these early events in their lives? An appeal to traces in answering these questions will not work for the simulationist because genuine remembering does not need a trace. The simulationist account of memory is too liberal in its ascriptions of remembering to explain the phenomenon of childhood amnesia.

## The Limits of the Personal Past

A further problem arises if the simulation theorist accepts these Dalí-type states as genuine memories, one that threatens to jeopardize the internal coherence of the theory. If the simulationist accepts that one can remember the moment of one’s birth or being in the womb, because one can (accurately) imagine such events, then what about cases that are even earlier in a human life? Can we remember the very early stages of gestation, or even the moment of conception, if we have access to information about how those moments unfolded (see, e.g., Duncan et al., 2016)? It seems that if remembering is merely an imaginative capacity, then the simulationist is forced to accept these as memories too. But, then, in such cases there is no (robust) subject of experience (Lagercrantz, 2014). This is where the problem arises for the simulation theory. Episodic remembering is representing events in one’s *personal* past. But, if the simulationist can remember such early events in their life, what happens to the “personal” in remembering the personal past? Attributing memory to imaginative representations of such early events, when there is no real sense that there is a subject of experience, means that the simulationist seems to have shifted from a first-personal to a third-personal notion of episodic memory: as long as there is access to the right information in the present, I can imagine the moment of another person’s birth just as well as my own, and they can imagine my birth just as easily as I can imagine it. But what is it, apart from a past subject of experience, that makes one imagining but the other remembering? Without a subject of experience, we seem to have lost the personal aspect of episodic memory.

The simulationist response here will be largely determined by their conception of the personal past. The simulationist wants to move away from a notion of the personal past that is defined in such a way that it relates to what one can experience. The simulationist prefers, rather, a notion of the personal past that relies “on our intuitive sense of what it is for a subject to be involved in an event,” where the personal past “can then be viewed as an ordered sequence of episodes in which the subject was involved” (Michaelian, 2016a, p. 107). This definition presupposes that there was a “subject” in the past, but there seems to be no sense in which these early events in our lives are episodes in which a *subject* was involved. Indeed, as Michaelian himself admits, episodic memory–with its connection to autonoetic consciousness, an awareness of the self traveling in subjective time–is intimately bound up with a robust sense of self, where this “sense of self turns out to be crucial to the ability to reliably remember the past” (Michaelian, 2016a, p. 6). That is part of what it means to episodically remember: to mentally travel back in time to a previous episode that the self experienced. For the simulationist, in episodic remembering:

I am aware of myself as the very subject who was involved in the event. It is in part due to this distinctive subjective dimension that it is not redundant to say that a subject *episodically remembers a given episode*: I can also semantically remember an episode, even an episode belonging to my personal past, but when I do so the retrieved representation typically lacks the feeling of warmth and intimacy which mark it as belonging to my personal past (Michaelian, 2016a, p. 214).

The key point is that even though imagination may require a present self to project into imaginary scenarios, episodic memory is thought to involve not only a robust sense of self in the present, but also a robust sense of self in the past. The key to episodic memory, and not imagination, is that there is a connection between these past and present selves.

This seems to leave the simulationist facing a dilemma. If he wants to deny that such very early events in our lives, such as the early weeks of gestation, can be remembered, then it seems that we need a new understanding of what it means to remember an event in the *personal* past. We seem to need a “subject” of experience. We are not successfully remembering these events because there was no robust subject of experience in the past. But then this makes it hard to see how we can remember non-experienced events, which is a key claim of the simulation theory.

If, on the other hand, the simulationist prefers to accept that we do not need a past subject of experience and that we *can* successfully remember such early events in our lives, then what is it that ensures that we are *episodically* remembering these events? Without defining the notion of “personal past” in terms of a diachronic subject of experience, the concept seems to have shifted to something more like a “life story.” There are two problems with this way of understanding the personal past. First, as we saw above, it could be argued that events in the lives of others (e.g., our parents, or ancestors) may be particularly important for our life story, so then the question arises as to why we cannot remember these events. If a past subject of experience is not necessary for successful remembering, then we have shifted substantially away from a first-personal understanding of episodic memory. If we can remember events that we (as subjects) were not involved in, then we seem a very short step away from a very different conception of what it means to episodically remember^16^. We seem to have lost the essential first-personal aspect of episodic memory.

Second, even if we can retain the first personal aspect of remembering events related to one’s life story, without referring to a subject of experience, are we still in the domain of episodic memory? If we can remember very early events in our “life story,” then we seem to be shifting from an account of episodic memory proper to something like “autobiographical memory.” Yet, the simulation theorist wants their account to be one of episodic memory not autobiographical memory. The reason for this is the idea that episodic memory is more likely to be a natural kind. Autobiographical memory, the simulationist tells us, is unlikely to qualify as a natural kind, but is rather “responsible for overall knowledge of one’s life history and is usually viewed not as the product of a single memory system but rather as a capacity emerging from the interaction of other, more fundamental capacities, including both episodic and semantic memory” (Michaelian, 2016a, p. 35). To explain memory of these very early events as belonging to autobiographical memory, however, is to go against the simulationist’s own “systems-based approach” to delineate and develop a naturalistic account of episodic memory. In these examples of representing moments from very early in our lives, we are trying to determine precisely whether these representations are *episodic memories* or not. Whether we can *episodically* remember such events is the key question, and it is unclear exactly what the simulationist response is.

## Concluding Remarks

The simulation theory is not supposed to be immune to every counterexample. Nonetheless, I think the issues that I have pointed out go deeper. They suggest that there are structural problems with the simulation theory. In order to account for the fact that we routinely forget in everyday contexts, and that we typically cannot remember events from our early childhoods, we need to invoke a causal connection to the past, one which is sustained by a memory trace. These features of human remembering reveal that personal memory is a diachronic capacity, and they disclose a problem with the core synchronic claims of the simulation theory.

Let us return to the epigraph with which I started this paper. W. G. Sebald asks the question “how do we remember, and what is it we find in the end?” (Sebald, 2001/2018). Part of the answer, I think, is that remembering is not just imagining. If remembering were simply imagining, we would be free, like Dalí, to recall any event in our personal past. But we cannot remember everything. The limits of imagining are not the same as the limits of remembering. Our imagination may be free, but our memories are constrained.

And, what is it we find in the end? Not simply a present imagining. But our previous experience of a past event.

## Author Contributions

The author confirms being the sole contributor of this work and has approved it for publication.

## Conflict of Interest

The author declares that the research was conducted in the absence of any commercial or financial relationships that could be construed as a potential conflict of interest.

## Footnotes

1 Throughout this paper I will be discussing personal (episodic) memory, which can be roughly understood as the (typically) imagistic recall of events in one’s personal past. This form of memory is thought to have a rich phenomenology, which is distinct to other forms of memory (e.g., semantic). It is sometimes thought to be crucially linked to narrative, but I take no stand on this issue here. See, for example, Tulving (1985), Hoerl (2007), and Keven (2016).

2 As we shall see below (see section “Remembering the Personal Past: Causal Connections, or Imaginative Simulations?”), there is an important difference between Michaelian’s and Hopkins’ views.

3 The terms “construction” and “reconstruction” are somewhat interchangeable and refer (broadly) to the creativity involved in remembering, such that each occurrence of a mental representation may be different and draw on various sources of information.

4 For the purposes of this paper, articulating the key idea of the causal theory is sufficient given that I am focusing on the simulation theory and its rejection of the necessity of an appropriate causal connection for successful remembering. For fuller accounts of the intricacies of the causal theory and the thought experiments used to develop its insights, see, for example, Martin and Deutscher (1966), Bernecker (2010), Robins (2016), Debus (2017), and Michaelian and Robins (2018).

5 The idea of continuous causal connection does not mean that the trace is always available; it is compatible with cases in which one can forget something for a period because the trace was not accessible. A continuous causal connection means rather that the trace is continuously stored between the original experience and the moment of retrieval (Michaelian, 2016a, p. 79).

6 The link between the original experience to the present memory via the trace means that the content of the memory will be sufficiently similar to the content of the experience. It may be that exact similarity is too strict, however, and minor changes in content are permissible. Even in everyday cases of remembering our memories do not preserve every detail or get everything right. A permissible change is typically thought to involve a *decrease* but not an *increase* in content (Bernecker, 2008, p. 155).

7 Hopkins (2018) also proposes that remembering is a form of imagining. On his account, however, remembering is imagining that is “controlled by the past.” The control that the past exerts over episodic memory stems from the fact that memories derive from past experiences. If there was no past experience, broadly construed, from which the current state derives, then it should not count as a memory (Hopkins, 2018, p. 49). In effect, Hopkins’ view can be construed as a version of the causal theory of memory (Michaelian, 2016a, p. 113), and so the problems facing the simulation theory that I develop in later sections do not apply to it.

8 There are other conditions (accuracy and internality) that the simulation theory invokes to provide a taxonomy of memory errors (Michaelian, 2016b). I discuss the internality condition in section “Recalling Non-experienced Events.” See Michaelian (2016b, 2018) for simulationist classifications of memory errors.

9 Of course, there is a sense in which any theory is going to see memory as a diachronic capacity because it is about present memories of past events. However, the term “synchronic” captures the idea that it is ultimately the context of retrieval, rather than the connection between encoding (and storage) and retrieval, that is important for the simulation theory.

10 Michaelian’s (2011b) paper on forgetting appeared before he developed the simulation theory; as I show, thinking about forgetting as involving the unavailability or inaccessibility of traces seems incompatible with simulationism. See also Tulving and Pearlstone (1966), who introduced the terms “availability” and “accessibility.”

11 Of course, there may also be *potentially* no forgetting on the causal theory, as long as one can access a memory trace. However, as I show below, the notion of a trace provides an elegant explanation of the phenomenon of forgetting, and this is not an explanatory manoeuver that is available to the simulationist. The simulationist cannot simply appeal to traces to account for forgetting.

12 On the influential scenario construction theory of episodic memory, a causal connection is necessary for genuine remembering, but may be maintained by a minimal trace, which is devoid of representational content (Cheng et al., 2016; Werning, 2020). Minimal traces, which store sparse sensory information, are combined with semantic knowledge at retrieval to construct a representation of the past. In effect, minimal traces, in a similar way to the simulation theory, mean that in an important sense the construction of content in episodic memory is a synchronic capacity, which occurs at the moment of retrieval. Whether some forms of forgetting, such as the forgetting of contextual details as memory becomes semanticized over time, can be explained by invoking minimal traces is an interesting question. It is a question that is beyond the scope of this paper, however, so I bracket it for now.

13 There are of course, as Caravà also notes, differences and tensions between the two theories. This solution to forgetting emphasizes forgetting’s active nature and competition between the sources used to construct the final memory representation, but whether it is acceptable to both enactivists and simulationists is going to depend on whether the simulationist appeals to internal representations or not. This is, at present, a commitment of the simulationist theory but not a necessary one (Michaelian and Sant’Anna, 2019).

14 There have been reports of direct recall of events during operations involving general anesthesia, “but these are rare and are nearly always attributable to faulty anesthetic technique or apparatus failure” (Andrade, 1995, p. 479). Even if there is no genuine explicit recall of events under anesthesia, there is some evidence that implicit learning, such as becoming familiar with words presented intra-operatively, can occur under anesthesia (e.g., Andrade, 1995). As I am not focusing on these cases I leave them aside.

15 On infantile amnesia, see, for example, Howe and Courage (1993) and Stylianopoulou and Stamatakis (2017).

16 See Pillemer et al. (2015) on vicarious memory, which is memory for the events in the lives of other people. See also Werning (2020) for how vicarious memory poses a problem for the simulation theory.
